# Impaired Long-Term Quantitative Cellular Response to SARS-CoV-2 Vaccine in Thiopurine-Treated IBD Patients

**DOI:** 10.3390/cells14151156

**Published:** 2025-07-26

**Authors:** Luis Mayorga Ayala, Claudia Herrera-deGuise, Juliana Esperalba, Xavier Martinez-Gomez, Elena Céspedes Martinez, Xavier Serra Ruiz, Virginia Robles, Ernesto Lastiri, Zahira Perez, Elena Oller, Candela Fernandez-Naval, Mónica Martinez-Gallo, Francesc Casellas, Natalia Borruel

**Affiliations:** 1 Unitat d’Atenció Crohn-Colitis, Servei d’Aparell Digestiu, Hospital Universitari Vall d’Hebron, CP 08035 Barcelona, Spain; 2 Servei de Microbiologia, Hospital Universitari Vall d’Hebron, Universitat Autònoma de Barcelona (UAB), CP 08035 Barcelona, Spain; 3 Servei de Medicina Preventiva i Epidemiologia, Hospital Universitari Vall d’Hebron, CP 08035 Barcelona, Spain; 4 Servei d’Immunologia, Hospital Universitari Vall d’Hebron, Vall d’Hebron Institut de Recerca (VHIR), Universitat Autònoma de Barcelona (UAB), CP 08035 Barcelona, Spain; 5 Centro de Investigación Biomédica en Red, Enfermedades Hepáticas y Digestivas, CIBEREHD, Instituto de Salud Carlos III, CP 28029 Madrid, Spain

**Keywords:** SARS-CoV2 vaccine, inflammatory bowel disease, cellular response, thiopurines

## Abstract

**Background**: Studies investigating the long-term cellular immune response to SARS-CoV-2 mRNA vaccines in patients with inflammatory bowel disease (IBD) remain limited, particularly among those receiving immunosuppressive therapy. **Methods**: We prospectively evaluated humoral and cellular immune responses at short-term (4–6 weeks) and long-term (6–12 months) time points following SARS-CoV-2 mRNA vaccination in patients with IBD receiving anti-TNF agents, thiopurines, or combination therapy. We defined the short-term response as the measurement taken 4–6 weeks after the second vaccine dose and the long-term response as the measurement taken between 6 and 12 months after the first determination. A cohort of healthy controls was included for short-term comparative analysis. **Results**: At long-term follow-up, quantitative humoral responses were reduced in patients receiving anti-TNF monotherapy. In contrast, a reduced quantitative cellular response was found in the thiopurine (median 0.7 UI/mL, *p* < 0.05) and anti-TNF combo groups (median 0.4 UI/mL, *p* < 0.01) compared to anti-TNF monotherapy (median 2.2 UI/mL). **Conclusions**: There was a robust long-term humoral and cellular response to vaccination, but a diminished quantitative cellular response in patients treated with thiopurines or combo therapy compared to anti-TNF monotherapy.

## 1. Introduction

The SARS-CoV-2 mRNA vaccine induces a robust humoral response in most patients with inflammatory bowel disease (IBD) [1]. However, the durability of this humoral response appears to be reduced in patients receiving treatment with anti-TNF, even after booster doses [2,3,4,5]. Furthermore, vaccine-induced antibody responses are attenuated and less durable, which appears to translate into a higher risk of breakthrough SARS-CoV-2 infection and reinfection with the new variants in patients treated with anti-TNF [6,7]. Most studies on IBD patients have focused on the humoral immune response by examining functional neutralizing antibody responses; although this functional neutralizing capacity reflects efficient protection against SARS-CoV-2 infection, it only partially informs on immunological protection. Beyond immunosuppressive therapy in IBD, several other clinical conditions and treatments have also been associated with impaired vaccine-induced immunity. Reduced humoral or cellular responses to SARS-CoV-2 vaccines have been reported in patients with diabetes, liver cirrhosis, and in those receiving immunomodulatory agents such as rituximab, mycophenolate mofetil (MMF), or methotrexate [8,9].

Large cohort studies have demonstrated that anti-TNF therapy, particularly when used in combination with thiopurine, significantly attenuates humoral responses to SARS-CoV-2 vaccination in patients with IBD. In contrast, short-term cellular immune responses appear to be largely preserved in patients with these treatments [2,3,7]. SARS-CoV-2-specific cellular immune responses are important for viral clearance, provide robust memory, mediate recognition of viral variants, and are at least equally important in combatting the infection, mostly when neutralizing antibody concentration decay [10]. Information on the cellular response after vaccination against SARS-CoV-2 in patients with IBD remains scarce. Previous studies primarily evaluating a short-term T cell response are inconsistent and with divergent results, mainly in those receiving anti-TNF treatment, as some studies showed reduced cellular responses [11,12,13,14], while others reported similar or even augmented T cell responses compared to healthy controls [5,15,16,17,18,19,20]. Additional real-world evidence is necessary to direct the prioritization of vaccines in the future. Our study aimed to evaluate the short- and long-term cellular responses to SARS-CoV-2 mRNA vaccines in a cohort of IBD patients receiving anti-TNF and thiopurine treatment.

## 2. Materials and Methods

### 2.1. Study Population and Data Collection

This was a prospective, observational study of IBD adult patients (≥18 years) with a confirmed diagnosis of ulcerative colitis (UC) or Crohn’s disease (CD), established using standard clinical, endoscopic, histological, and radiological criteria. Patients were required to have been on stable treatment for at least six months with either anti-TNF agents (adalimumab, infliximab, or golimumab, as monotherapy or in combination with thiopurines) or thiopurine monotherapy (azathioprine or 6-mercaptopurine). Individuals receiving other immunosuppressive agents or with other chronic comorbidities unrelated to IBD were excluded. We also included a group of healthy health worker volunteers.

All participants received at least two doses of an mRNA SARS-CoV-2 vaccine (BNT162b2 [Pfizer-BioNTech, New York, NY, USA] or mRNA-1273 [Moderna, Cambridge, MA, USA]) as per local guidelines. The initial scheme included two vaccine doses administered 28 days apart during April and May 2021; a subsequent third and fourth dose were recommended between October 2021 and January 2022 and between May and July 2022, respectively. Blood samples were obtained from all participants at a minimum of two time points, short-term and long-term, for evaluation of the immune response to vaccination, with the exception of healthy controls, who were assessed only at the short-term time point. We defined the short-term response 4–6 weeks after the second vaccine dose and the long-term response as the measurement between 6 and 12 months after the first determination. The study was approved by the Ethics Committee for Research with Medications (CEIm) of Vall d’Hebron University Hospital (CEIm Code: EOM(AG)030/2021(5831)). All participants provided written informed consent in accordance with the Helsinki Declaration. All authors had access to the study data and reviewed and approved the final manuscript.

### 2.2. Assessment of Humoral Immune Response

The serological response to SARS-CoV-2 was determined by detecting specific antibodies against nucleocapsid and spike SARS-CoV-2 antigens. Two commercial chemiluminescence immunoassays (CLIAs) were used: (1) Elecsys^®^ Anti-SARS-CoV-2 (Roche Diagnostics, Mannheim, Germany) performed on the Cobas^®^ 8800 system (Roche Diagnostics, Rotkreuz, Switzerland) for the determination of the total antibodies (including IgG, IgM, and IgA) against nucleocapsid SARS-CoV-2 proteins (IgG-N) (cut-off = 1.0 index), and (2) LIAISON^®^ SARS-CoV-2 TrimericS IgG (DiaSorin, Stillwater, MN, USA) performed on the LIAISON^®^ XL Analyzer (DiaSorin, Saluggia, Italy) for the determination of IgG antibodies against the spike (IgG-S) glycoprotein of SARS-CoV-2 (range <4.81 to >2080 BAU/mL. A positive humoral response or seroconversion was defined when an IgG-S titer was ≥260 BAU/mL based on the agreement to promote an international clinically relevant standard measurement unit [21]. We calculated the seroconversion rate as the percentage of patients with a determination over the cut-off value.

### 2.3. Assessment of Cellular Immune Response

The SARS-CoV-2-specific T cell response was assessed via whole-blood Interferon-Gamma Release immuno-Assay (IGRA) technology using QuantiFERON^®^ SARS-CoV-2 RUO tubes from Qiagen^®^ (Hilden, Germany), consisting of two tubes containing proprietary mixes of SARS-CoV-2 spike protein (S1, S2, RBD), and one positive and one negative control tube. This test was used following the manufacturer’s instructions. Briefly, venous blood samples were collected directly in the four QuantiFERON^®^ SARS-CoV-2 RUO tubes, incubated at 37 °C for 16–24 h, and centrifuged to separate plasma. IFN-γ (IU/mL) was measured via CLIA using the LIAISON^®^ QuantiFERON-^®^TB Gold Plus assay (DiaSorin, Saluggia, VC, Italy) on the LIAISON^®^ XL Analyzer (DiaSorin, Italy). We used experimentally established cut-off values (Ag1 = 0.051 and Ag2 = 0.442) for the qualitative interpretation of the results [22].

### 2.4. Clinical Variables in Inflammatory Bowel Disease, Vaccine Effect, and COVID-19 Infection

We collected the disease activity data from the Simple Clinical Colitis Activity Index (SCCAI) in UC and Harvey-Bradshaw (HBI) in CD patients, questionnaires which were self-filled at baseline, 14–21 days after the second dose of vaccine and in the long-term evaluation time points. Adverse events (AEs) related to the vaccine were recorded using a symptom questionnaire after the first and second doses. The incidence of COVID-19 infection and the hospitalization rate after the vaccine doses were collected. We considered a previous SARSCoV-2 infection if the subject had a positive IgG-N.

### 2.5. Statistical Analysis

No formal sample size calculation was performed. Given the observational and exploratory nature of this study on SARS-CoV-2 mRNA vaccine responses, and the lack of prior data on the expected effect sizes or variability in long-term immune responses, we included all eligible participants during the study period. We performed a descriptive analysis of variables. Continuous variables were expressed as median and categorical as absolute values. Non-parametric tests were used to assess associations between clinical factors and immune response. For comparisons between two groups, Fisher’s exact or Mann–Whitney U test was used. For more than two groups, the Kruskal–Wallis test was used. Multiple comparisons corrections were performed using Dunn’s test. Paired cellular and humoral responses between the two time points were compared using the Wilcoxon signed-rank test. Differences in long-term IFN-γ (IU/mL) levels between treatment groups were assessed using a univariate general linear model, adjusting for covariates. Pairwise comparisons were conducted using Bonferroni-adjusted estimated marginal means. A 2-tailed *p* value < 0.05 was considered significant.

## 3. Results

From 1 June 2021 to 31 August 2022, 166 participants were recruited, 148 IBD patients and 18 HC patients (Table 1). Patients were divided into three groups: (1) IBD patients receiving anti-TNFα monotherapy (*n* = 57); (2) patients receiving anti-TNFα therapy in combination with thiopurines (*n* = 53); and (3) patients receiving thiopurine monotherapy (*n* = 38). In the thiopurine group, 82% were treated with azathioprine and 18% with mercaptopurine. All patients in the combination anti-TNFα therapy group received azathioprine.

Subjects received a median of three vaccines. No serious adverse events or disease flares were reported. Patients infected with SARS-CoV-2 presented with mild disease and only one patient on anti-TNFα was briefly hospitalized.

### 3.1. Humoral Immune Response

**Figure 1:**
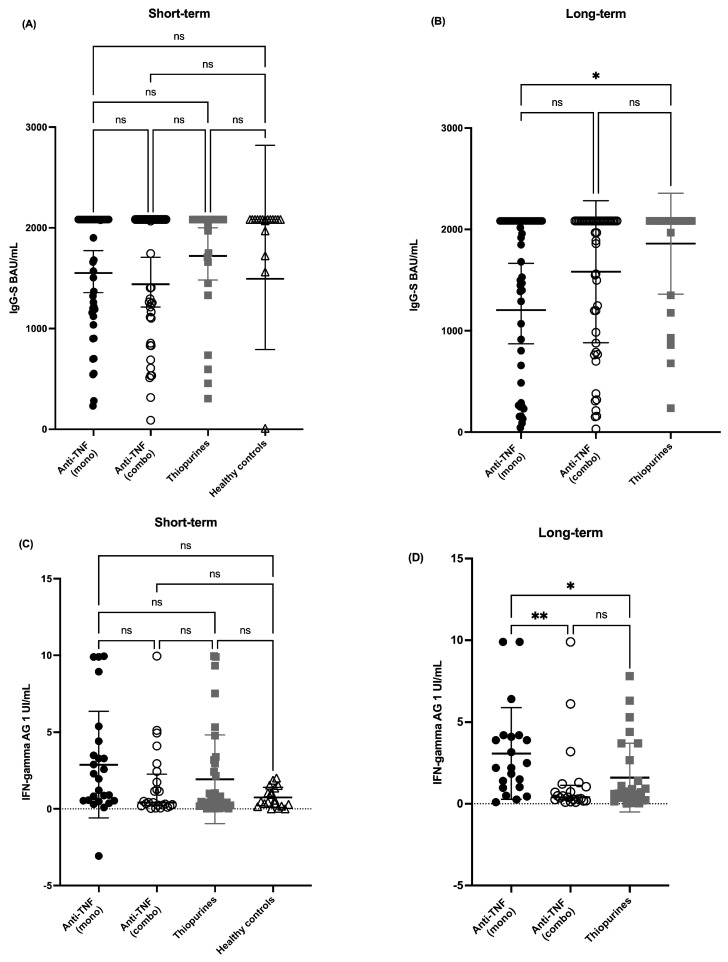
Short- and long-term quantitative humoral and cellular immune responses to SARS-CoV-2 mRNA vaccination between groups. (**A**) Short-term IgG-S median concentrations. (**B**) Long-term IgG-S median concentrations. (**C**) Short-term AG1-NIL median concentrations. (**D**) Long-term AG1-NIL median concentrations. * *p* < 0.05, ** *p* < 0.01.

After a median of 4 weeks and two vaccines, 97.8% of patients achieved seropositivity: 98% in the anti-TNF monotherapy group, 96% in the combo group, and 100% in the thiopurine group. Healthy subjects had a 100% seropositivity rate, with no differences in short-term IgG-S concentration (Figure 1A).

In the long term (median 20 weeks, 3 vaccines), 91% overall seropositivity was achieved: 85% in the anti-TNF monotherapy group, 92% in the combo group, and 97% in the thiopurine group (*p* = ns). Anti-TNFα monotherapy was associated with a lower IgG-S concentration (median 2050 (887–2082)) compared to the thiopurine group (median 2082 (1240–2082), *p* < 0.05), with no significant differences between anti-TNF monotherapy and combo therapy groups (Figure 1B and Appendix Table A1). At long term, 44.5% were anti-N positive. Previous COVID-19 infection was associated with higher antibody titers (2082 (1915–2082) vs. 1523 (529–2082), *p* < 0.001).

To assess changes in humoral immune responses over time, we compared short-term and long-term measurements. No significant differences were observed across time points for the anti-TNF monotherapy group (*p* = 0.31), the thiopurine group (*p* = 0.86), or the combination therapy group (*p* = 0.41).

Overall, 91% of patients maintained long-term positive humoral responses. However, anti-TNF monotherapy patients showed a decreased long-term positivity rate (98% short-term vs. 85% long-term, *p* < 0.05). IgG-S median concentrations were similar between time points. Two patients were IgG-S negative in the short term and seroconverted in the long term, while one remained negative despite four vaccine doses. Twelve patients, initially positive in the short term, lost the humoral response and were IgG-N negative. Of these, seven were in the anti-TNF monotherapy group and four in the combo group.

### 3.2. Cellular Immune Response

In the short-term evaluation, 89% of the cohort achieved a positive cellular immune response: 92% in the anti-TNF monotherapy group, 88% in combo therapy, 87% in the thiopurine group, and 84% of healthy controls. There were no IFN Gamma concentration differences (median AG1: 1.86 UI/mL; AG2: 1.50 UI/mL) between groups (Figure 1C and Table 2). In the long term, 97% of patients showed a cellular response, with 100% positivity in both the anti-TNF monotherapy and combo groups, and 93% in the thiopurine group (*p* = ns). However, anti-TNF monotherapy patients had significantly higher IFN Gamma concentrations (median 2.2 UI/mL (1.0–4.15)) compared to the anti-TNF combo (median 0.4 UI/mL (0.22–1.13); *p* < 0.01) and thiopurine groups (median 0.7 UI/mL (0.26–2.35); *p* < 0.05) (Figure 1D). No significant association was found between long-term cellular response and type of IBD, disease duration, number of vaccines, vaccination interval, or history of previous infection.

To assess changes in humoral immune responses over time, we compared short-term and long-term measurements. No significant differences were observed across time points for the anti-TNF monotherapy group (*p* = 0.84), the thiopurine group (*p* = 0.28), or the combination therapy group (*p* = 0.50).

Cellular response persistence was observed in 98% of patients, both qualitatively and quantitatively. One patient, on thiopurines, changed from positive short term to negative long term despite two vaccine doses with good humoral response. Of eight patients without a short-term cellular response, seven achieved long-term positivity, and one remained negative after three doses.

A general linear model, adjusting for age, sex, type of IBD, and number of vaccines, was used to assess differences in long-term IFN-γ (IU/mL) levels among the three treatment groups: anti-TNF monotherapy, combo, and thiopurine. The model revealed a statistically significant overall group effect (F (2, 66) = 3.27, *p* < 0.01), with a moderate effect size (partial η^2^ = 0.090), indicating that antibody responses differed between at least two of the groups.

Post hoc pairwise comparisons using Bonferroni correction demonstrated significantly higher long-term IFN-γ (IU/mL) levels in the antiTNF monotherapy group compared to thiopurine (mean difference = 1.76, *p* < 0.05, 95% CI: 0.09–3.60). Differences between antiTNF monotherapy and the antiTNF combo (mean difference = 1.50, *p* = 0.113) and between combo and thiopurine groups (mean difference = 0.26, *p* = 1.000) were not statistically significant.

## 4. Discussion

Vaccination against SARS-CoV-2 is crucial for preventing severe disease. A large UK study found that patients with immune-suppressive conditions who had lower humoral and cellular responses after two vaccine doses were associated with hospitalization or death from COVID-19 [23].

We observed a robust short-term cellular response with no differences between cohort subjects. Furthermore, IBD patients showed sustained long-term responses, with an 8% increase in positivity, suggesting booster benefits. Although long-term response rates were similar across treatment groups, thiopurine-treated patients had lower cellular concentrations than those on anti-TNF monotherapy. The clinical relevance of this is uncertain, but it may be valuable for patients and healthcare providers. Despite robust and long-lasting immune responses, some patients experienced a decline in long-term responses. Although no standardized cellular immune correlates of protection have yet been established for SARS-CoV-2, several studies suggest that vaccine-induced T cell responses, particularly IFN-γ–producing CD4^+^ and CD8^+^ T cells, play an important role in mitigating disease severity, especially when antibody levels wane [24,25]. In this context, IFN-γ release assays have been proposed as functional markers of vaccine-induced cellular immunity and may complement serological data to assess immunological protection [26,27]. Moreover, recent human data have shown that prior vaccination promotes the early activation of memory T cells upon SARS-CoV-2 breakthrough infection, leading to enhanced immune responses and early viral control [28]. This further supports the clinical relevance of vaccine-induced T cell immunity, even in the absence of neutralizing antibodies, and reinforces the value of IFN-γ as a measurable correlate of protection, despite the current lack of defined quantitative thresholds.

Previous studies have investigated the short-term cellular immune response to SARS-CoV-2 vaccination in IBD patients receiving anti-TNF therapy, with heterogeneous findings. Some have reported impaired T cell responses under TNFα blockade, including reduced IFN-γ production and diminished T cell proliferation, suggesting both quantitative and qualitative defects in cellular immunity [11,12,13]. For example, Geisen et al. and Cerna et al. observed attenuated cellular responses in anti-TNF-treated patients, particularly in the absence of booster doses. In contrast, other studies have demonstrated preserved—or even enhanced—cell-mediated immune responses in patients receiving anti-TNF therapy, especially following additional vaccine doses [14,15].

Two studies have evaluated cellular immune response after a third vaccine dose. The VIP study reported that most infection-naive IBD patients treated with thiopurines, infliximab, ustekinumab, or vedolizumab exhibited T cell responses comparable to healthy controls [4]. The STAR SIGN study assessed both humoral and cellular responses after a third vaccine dose in IBD patients receiving biologics and healthy controls, and found lower cellular responses in anti-TNF patients [14]. Caldera et al. analyzed the cellular response to vaccination in IBD patients, primarily those receiving biologic therapy, six months post third dose. In line with our findings, a robust cellular response at all time points was observed, and anti-TNF monotherapy patients had a higher cellular IFN-γ concentration compared to those on non-anti-TNF therapy [29]. The mechanism underlying this enhanced response to anti-TNF therapy is unclear, but TNF-alpha might influence vaccine-induced humoral and cellular responses by supporting B-cell maturation and limiting T cell expansion. This inhibition of TNF-α could therefore augment cellular responses and reduce antibody levels in anti-TNF patients [30].

The altered cellular response to mRNA vaccines in thiopurine-treated patients is also unclear, but may be explained by their known immunosuppressive mechanisms. Azathioprine and its active metabolite 6-mercaptopurine inhibit de novo purine synthesis, thereby limiting lymphocyte proliferation. Moreover, they induce apoptosis of activated CD4^+^ T cells and inhibit Rac1-mediated signaling pathways, including NF-κB and STAT3, which are critical for T cell survival and expansion upon antigen stimulation [31]. Other studies have shown that treatment with thiopurines might result in the depletion of antigen-specific T memory cells, which rely on repeated encounters with the antigen [32]. These mechanisms may underlie the diminished IFN-γ production we observed in this patient subgroup.

While our primary focus was on cellular immunity, we also examined humoral responses in parallel. Interestingly, our findings on short-term antibody responses in anti-TNF-treated patients differed from those in previous reports. Macaluso et al. [33] observed significantly lower seropositivity and antibody levels in this group compared to healthy controls. This discrepancy may reflect differences in study design, sample size, or vaccine timing; notably, their assessment occurred at eight weeks post vaccination, while ours was conducted earlier (4–6 weeks), potentially capturing different stages of the antibody response. Moreover, our smaller control group and limited long-term follow-up represent additional limitations. Nevertheless, in our extended follow-up, anti-TNF monotherapy was associated with reduced IgG-S concentrations relative to thiopurine-treated patients, though no significant differences emerged when compared to combination therapy.

Our study has several strengths. First, we evaluated the cellular immune response using a time-efficient and easily interpretable assay, allowing for practical integration into real-world clinical and research workflows. This approach provides meaningful insights into T cell-mediated immunity, which is particularly relevant for understanding long-term protection against SARS-CoV-2 in immunosuppressed populations. Second, we selected patients receiving stable maintenance regimens of immunosuppressive therapies—specifically anti-TNF agents, thiopurines, or combination therapy—reflecting common treatment patterns in contemporary IBD management. This enhances the clinical applicability of our findings, as it allowed us to assess vaccine-induced immune responses in a well-defined and representative population. Moreover, the inclusion of both humoral and cellular endpoints offers a more comprehensive view of the immune landscape across different therapeutic exposures.

There are also a few limitations. These include the relatively small overall cohort size and the absence of a formal sample size calculation. At the time of study design, there were no robust data available to inform a priori estimates of effect size or variability in long-term immune responses to SARS-CoV-2 vaccination in IBD patients receiving immunosuppressive therapies. As a result, subgroup sizes were determined via feasibility and real-world treatment distributions. While the study provides important exploratory insights, the limited sample size—particularly in some subgroups for cellular outcome analyses—may have reduced our ability to detect smaller or more nuanced differences. This limitation should be considered when interpreting the findings.

In addition, long-term follow-up data were not available for the healthy control group. These participants were recruited during the early phase of the vaccination campaign for short-term comparative analysis only and were not scheduled for extended follow-up due to logistical constraints. The control cohort was also limited in size, with only 18 individuals included, reflecting the availability of matched participants under standardized sampling conditions. While this restricted our ability to perform robust long-term comparisons between patients and controls, the primary focus of the study was to evaluate sustained immune responses in immunosuppressed IBD patients, a population for whom vaccine durability carries greater clinical relevance.

In conclusion, we found a sustained and robust long-term humoral response to vaccination, but a diminished quantitative cellular response in IBD patients treated with thiopurines or combination therapy compared to those receiving anti-TNF monotherapy. These findings suggest that patients on these therapies may have altered long-term cellular immunity, highlighting the need for further studies to confirm these observations and clarify their clinical implications, particularly in relation to booster vaccination strategies.

## Figures and Tables

**Table 1:** Baseline characteristics of study participants by treatment group.

*n* = 166	Anti-TNF mono *n* = 57	Anti-TNF combo *n* = 53	Thiopurines *n* = 38	Controls *n* = 18	*p*
**Age** (mean ± SD)	45 ± 13.96	43 ± 11.26	40 ± 10.77	51 ± 11.87	*p* = 0.06
**Gender** *n* (% men)	32 (56)	31 (58)	26 (68)	5 (28)	*p* = 0.025
**Years of diagnosis** (mean ± SD)	12 ± 8.77	13 ± 8.23	10,5 ± 9.5	—	*p* = 0.43
**Crohn’s disease/Ulcerative colitis** (*n*)	47/10	39/14	17/21	—	*p* < 0.01
**Anti-TNF** *n* (%) Infliximab Adalimumab Golimumab	18 (32) 37 (65) 2 (3)	32 (60) 20 (38) 1 (2)	—	—	*p* < 0.05
**Months of treatment** (mean ± SD)	64 ± 53.93	77 ± 51.73	105 ± 74.86	—	*p* = 0.07
**Escalated dose** (%)	27 (47)	23 (43)	—	—	*p* = 0.35
**Simple Colitis Activity Index** (mean ± SD)	2 ± 3.53	0 ± 1.94	1 ± 1.16	—	*p* = 0.18
**Harvey-Bradshaw** (mean ± SD)	0 ± 1.88	1 ± 1.58	0 ± 1.77	—	*p* = 0.72

—Data not available for this group.

**Table 2:** Quantitative cellular immune responses at short- and long-term time points by treatment group.

	Anti-TNF mono	Anti-TNF combo	Thiopurine	Controls	
**AG1 UI/mL Short-term**	1.36 (0.53–3.95)	0.51 (0.20–2.26)	0.66 (0.24–2.68)	0.56 (0.18–1.35)	*p* = 0.051
**AG2 UI/mL Short-term**	1.03 (0.36–2.79)	0.39 (0.14–1.39)	0.45 (0.15–1.45)	0.61 (0.17–1.97)	*p* = 0.26
**AG1 UI/mL Long-term**	2.2 (1.0–4.15) *^#^	0.4 (0.22–1.13) *	0.7 (0.26–2.35) ^#^	—	* *p* < 0.01 ^#^ *p* < 0.05
**AG2 UI/mL Long-term**	1.51 (0.66–4.27)	0.23 (0.11–0.59)	0.51 (0.16–1.09)	—	*p* = 0.08

*^#^ Groups with statistically significant differences are shown.

## Data Availability

Data sets of the current study are available from the corresponding author on request.

## Abbreviations

The following abbreviations are used in this manuscript:

AE	Adverse Event
AG1/AG2	Antigen Tube 1/Antigen Tube 2
BAU/mL	Binding Antibody Units per Milliliter
CD	Crohn’s Disease
CEIm	Comité de Ética de Investigación con Medicamentos
CLIA	Chemiluminescence Immunoassay
COVID-19	Coronavirus Disease 2019
HC	Healthy Controls
HBI	Harvey-Bradshaw Index
IBD	Inflammatory Bowel Disease
IFN-γ	Interferon Gamma
IGRA	Interferon Gamma Release Assay
IgG-N	Immunoglobulin G against Nucleocapsid
IgG-S	Immunoglobulin G against Spike protein
IU/mL	International Units per Milliliter
mRNA	Messenger Ribonucleic Acid
PCR	Polymerase Chain Reaction
RBD	Receptor Binding Domain
RN	Registered Nurse
SARS-CoV-2	Severe Acute Respiratory Syndrome Coronavirus 2
SCCAI	Simple Clinical Colitis Activity Index
SD	Standard Deviation
TNF	Tumor Necrosis Factor
UC	Ulcerative Colitis
VHIR	Vall d’Hebron Institut de Recerca

## Appendix A

**Table A1:** Quantitative humoral immune responses at short- and long-term time points by treatment group.

	Anti-TNF mono	Anti-TNF combo	Thiopurine	Controls	
**IgG-S BAU/mL Short-term**	2082 [1261–2082]	2082 [1189–2082]	2082 [1915–2082]	2082 [2040–2082]	*p* = 0.26
**IgG-N BAU/mL Short-term**	0.09 [0.08–0.13]	0.09 [0.08–0.09]	0.09 [0.08–0.4]	0.07 [0.07–1.36]	*p* = 0.07
**IgG-S BAU/mL Long-term**	2050 [887–2082] *	2038 [985–2082]	2082 [1240–2082] *	NA	* *p* < 0.05
**IgG-N BAU/mL Long-term**	0.31 [0.08–5.26]	0.47 [0.09–9.01]	0.35 [0.1–6.20]	NA	*p* = 0.25

* Groups with statistically significant differences are shown.
